# The Effects of Body Cold Exposure (Cryolipolysis) on Fat Mass and Plasma Cholesterol

**DOI:** 10.3390/life14091082

**Published:** 2024-08-29

**Authors:** Rodrigo Alvaro Brandão Lopes-Martins, Ludymilla Vicente Barbosa, Mirian Martins Barbosa Sousa, Anna Beatriz Lobo, Elize Leonice da Rocha Santos, Alberto Souza de Sá Filho, Matheus Bernardes Souza, Jivago Carneiro Jaime, Constanza Thaise Xavier da Silva, Carlos Ruiz-Silva, Patrícia Sardinha Leonardo

**Affiliations:** 1 Laboratory of Biophotonics and Experimental Therapeutics (LABITEX), Universidade Evangélica de Goiás, Av. Universitária Km 3,5, Anápolis 75083-515, Brazilloboannabeatriz08@gmail.com (A.B.L.); matheusmbs19@hotmail.com (M.B.S.); constanzathaise@yahoo.com.br (C.T.X.d.S.); 2 Programa de Pós-Graduação em Bioengenharia, Universidade Brasil, Av. Carolina Fonseca 236, Itaquera, São Paulo 08230-030, Brazil; 3 Laboratory of Health Technologies (LATES), Universidade Evangélica de Goiás, Av. Universitária Km 3,5, Anápolis 75083-515, Brazil; patyssardinha@gmail.com (L.V.B.); elise.santos@unievangelica.edu.br (E.L.d.R.S.); patricia.martins@unievangelica.edu.br (P.S.L.); 4 Department of Physical Education, Evangelical University of Goiás (UniEVANGÉLICA), Anápolis 75083-515, Brazil

**Keywords:** cryolipolysis, body fat reduction, plasma lipid profile, non-invasive body contouring, metabolic health, obesity, cardiovascular risk factors, therapeutic cryotherapy

## Abstract

Introduction: This study investigates the impact of cryolipolysis on reducing localized fat and altering plasma lipid profiles in 30 overweight and obese women. Conducted at the Health Technology Laboratory of the Evangelical University of Goiás, this clinical research adhered to stringent ethical guidelines. Methods: Participants underwent three cryolipolysis sessions, with comprehensive assessments of body composition and plasma lipids performed pre- and post-intervention. Results: Significant findings include a reduction in abdominal fat mass by an average of 4.1 kg and a decrease in BMI by 0.7 points (*p* < 0.05). Notably, total cholesterol levels decreased by an average of 15.7 mg/dL, and LDL cholesterol saw a reduction of 10.2 mg/dL (*p* < 0.01), with no significant changes in HDL cholesterol or triglyceride levels. These results suggest that cryolipolysis, in conjunction with standardized dietary control, offers a non-invasive alternative to surgical fat reduction, potentially mitigating cardiovascular risks associated with obesity. Conclusions: The study confirms the efficacy of cryolipolysis in targeted fat reduction and underscores its role in improving key cardiovascular risk factors. These findings warrant further exploration into the long-term benefits of cryolipolysis in metabolic health management and not only for aesthetic treatments.

## 1. Introduction

According to a recent report by the World Health Organization (WHO), in 2016, 39.1% of adults globally were overweight, with 13.2% (over 650 million individuals) classified as obese—a figure approximately three times higher than in 1975 [1]. Obesity significantly elevates the risk of various chronic diseases, including diabetes mellitus and cardiovascular diseases (CVD), particularly heart disease and stroke [1]. While obesity prevalence among adults is slightly higher in women than in men, the incidence and severity of CVD are lower in premenopausal women and increase post-menopause [2,3]. Several factors contribute to this disparity between sexes in cardiovascular disease and metabolic risks, including sex hormones, sex chromosomes, physical activity, smoking, and environmental factors [4].

The escalating global prevalence of obesity represents a complex health crisis with profound implications that extend beyond physiological effects to significant aesthetic concerns. The increasing rates of obesity not only compromise individual health and well-being but also pose aesthetic challenges [5,6]. Excessive adiposity alters body proportions and contours and fosters societal tendencies towards unrealistic beauty standards, exacerbating body image dissatisfaction and contributing to a culture of appearance-focused discrimination. The normalization of overweight and obesity further blurs the distinctions between healthy and unhealthy body weights, perpetuating misconceptions about ideal aesthetics and reinforcing harmful lifestyle behaviors. Thus, addressing the obesity epidemic requires a comprehensive approach that targets both physiological health and the complex interplay between societal perceptions of beauty and body image [6,7,8,9].

Localized fat accumulation poses significant aesthetic and health concerns today [6]. This condition typically results from uncontrolled increased caloric intake and reduced energy expenditure and may be associated with the development of cardiovascular diseases, compromised body measurements, and aesthetic discomfort [7,8,9].

Liposuction is widely regarded as the primary method for removing excess adipose tissue. According to the Aesthetic Society, there was a notable 68% surge in the number of liposuction procedures in the United States between 2020 and 2021 [10]. Despite its popularity, alternative noninvasive body contouring techniques, such as cryolipolysis, are gaining global attention. These techniques include radiofrequency, laser, ultrasound, injectables, and thermal energy-based treatments. Cryolipolysis, in particular, accounted for more than a quarter of the over 1 million body sculpting procedures performed in 2019, as reported by the American Society for Dermatologic Surgery [10,11,12].

Cryolipolysis utilizes selective and controlled cooling based on the principle that lipid-rich tissues are more susceptible to cold damage than surrounding water-rich tissues. These deceased cells trigger an inflammatory response and are metabolically eliminated through phagocytosis, a natural digestive process of the body, leading to reduced fat deposition in the treated area [13,14].

Technological advancements have led to various treatments to address localized fat, with cryolipolysis standing out as a non-invasive method for body contouring without damaging subcutaneous tissue [15]. Initially validated as a standalone technique for reducing localized fat through controlled freezing, cryolipolysis has been enhanced with adjunctive approaches such as contrast cryolipolysis, which incorporates heating periods at the beginning or end of the application, yielding promising outcomes. Additionally, cryolipolysis has been combined with other therapeutic modalities to promote lipolysis and enhance efficacy [16].

Adipose tissue plays a central role in regulating energy balance, serving as an energy reservoir primarily controlled by lipoprotein lipase and lipogenic enzymes. Lipolysis, the release of fatty acids from adipose tissue, is predominantly regulated by catecholamines such as noradrenaline and adrenaline during energy demand. This process relies on adrenergic receptors, particularly β-adrenoceptors, which may exhibit dysfunction in obesity, potentially resulting in reduced fatty acid release during energy-demanding situations such as exercise and other stress situations [17].

Cold temperatures induce physiological stress by triggering vasoconstriction, reducing blood flow to the skin and extremities, which can lead to decreased tissue oxygenation and an increased risk of frostbite. Exposure to cold activates the body’s thermoregulatory mechanisms, including shivering and increased metabolism, in an effort to maintain core body temperature, placing additional strain on metabolic pathways and energy reserves. Prolonged exposure to cold can disrupt hormonal balance, including increased secretion of stress hormones such as cortisol and adrenaline, contributing to heightened physiological stress responses [18]. In this context, cryolipolysis has become a popular treatment for non-invasively addressing localized fat accumulation, circumventing the risks associated with reconstructive surgeries, such as anesthesia-related complications, bleeding, contour irregularities, scarring, altered sensitivity, infections, or fat embolism [19].

The bioelectrical impedance analysis (BIA) method has become extremely popular for assessing body composition because it is an easy-to-use, portable, quick, relatively inexpensive, and non-invasive technology. Consequently, BIA is widely used in hospitals, clinics, and other healthcare facilities [20,21].

This study seeks to assess the impact of plate cryolipolysis on reducing localized body fat, specifically targeting abdominal regions. Thus, our objective is to study the effects of cryolipolysis on fat mass and plasma lipids in overweight and obese patients.

## 2. Materials and Methods

This clinical study aimed to evaluate the efficacy of plate cryolipolysis in promoting lipolysis in the abdominal and flank regions of women with localized fat deposits. The research was conducted at the Health Technology Laboratory of the Evangelical University of Goiás and adhered to the Guidelines and Regulatory Norms for research involving human beings, as established by the National Health Council, Ministry of Health, in October 1996 and updated in Resolution 466/2012, Brazil. This study received approval from the Ethics Committee of the Evangelical University of Goiás—UniEVANGÉLICA under the registration number 6.574.522 from 23 December 2023.

### 2.1. Sample Calculation

Thirty female participants, aged between 18 and 50 years, were invited to participate in this study. The sample size of 30 participants was determined through a statistical calculation using G*Power 3.1.9.7 software, based on a paired T-test correlation: point biserial model with a power of 0.90, an alpha (α) of 0.05, and an effect size of 0.4. The calculation indicated that a minimum of 28 participants would be required. To account for potential dropouts or withdrawals during the intervention, a total of 30 participants was included.

### 2.2. Data Collection

Participants were recruited at the university campus without distinction of activities and provided informed consent before participating in the study. Initial assessments included a detailed body history to evaluate lifestyle habits and medical history, classification of body biotype, abdominal diastasis testing, body composition analysis using bioimpedance with a BIA device (Tera Science™, São José dos Campos, Brazil), infrared thermography with a FLIR Thermographic Camera (FLIR—Stockholm, Sweden), and anthropometric measurements using a standard tape measure. Blood analyses for lipids, triglycerides, and ferritin were also conducted. Standardized photography before and after each of the three cryolipolysis sessions, spaced 15 days apart, was also recorded for comparisons. For the photos, participants were positioned in anterior, posterior, right profile, and left profile positions. The distance observed for each image was 70 cm, and participants signed an authorization form for the use of the images. All parameters were compared before and after three sessions, and each individual was compared to itself using the paired student *t*-test.

### 2.3. Cryolipolysis

Cryolipolysis treatment was administered using four simultaneous handles for 40 min at a temperature of −5 °C, utilizing Asgard Equipment (Adoxy™—Votorantim, Brazil). Standard antifreeze blankets were employed to protect the treated areas. Each session lasted 40 min, with intervals of 15 to 20 days between sessions. The procedure adhered to the manufacturer’s guidelines, placing the four handles on the abdominal region, followed by a gentle reperfusion massage post-treatment. Infrared thermography analysis was used to monitor blood flow restoration.

Participants were positioned supine during the cryolipolysis procedure, with the four device handles placed on the abdominal region. After the procedure, a reperfusion massage was administered to facilitate the return to normal body temperature, with continuous monitoring every 5 min using a C5 infrared thermography camera (FLIR). Subsequently, participants completed a Likert scale questionnaire to assess satisfaction levels before and after cryolipolysis.

### 2.4. Computerized Bioimpedance Analysis (BIA—Tera Science, Brazil)

Computerized bioimpedance analysis is a method used to assess body composition. The computerized version of this test offers a more detailed and precise analysis, allowing data to be easily interpreted and monitored over time. This is particularly useful in clinical and fitness contexts to monitor changes in body composition and adjust treatment or training plans. It is a non-invasive, quick, and relatively easy method to use, although its accuracy can be affected by factors such as hydration, food intake, and recent physical activity.

Specifically, the phase angle (PhA) is a useful indicator of cell membrane integrity, the distribution of water between intracellular and extracellular spaces, and the prediction of body cell mass, as they are described by the components of electrical impedance (Z): resistance (R; a function of the volume of intracellular and extracellular fluid) and reactance (Xc; a function of the dielectric material of tissue cells). The PhA is geometrically calculated from R and Xc measured at 50 kHz. The phase angle (PhA) can be simply calculated as an arctangent using the raw data of R and Xc at a frequency of 50 kHz, as follows: (Xc/R) × 180°/π. Thus, the PhA is obtained directly from the BIA without using a regression equation.

### 2.5. Preparation for Bioimpedance Testing

The participants receive instructions that they should fast for generally 4 to 6 h before the test. It was also recommended to avoid excessive water intake immediately before the test but maintain normal hydration in the 24 h prior. To avoid strenuous exercise in the 12 h prior to the test and avoid consumption of alcohol and caffeine 24 h before the test.

At the time of the test, the participants were kept on their feet with their bodies relaxed and their limbs slightly separated from the torso. Electrodes were typically placed on the wrists and ankles. The skin where the electrodes are applied was cleaned and dried before fixing the electrodes. Then, the electrodes were connected to wires, which, in turn, were connected to the bioimpedance device (BIA—Tera Science, Brazil).

Once the electrodes are correctly positioned and connected, the machine is activated. A small, typically imperceptible electric current is sent through the electrodes. This current quickly travels through the body from the lower to the upper limbs. Results were automatically calculated by the package.

### 2.6. Blood Collection

Blood samples were taken before the first session and again after the third session for plasma lipid profile analysis. Blood samples were collected from participants after an overnight fast. Plasma lipid levels, including total cholesterol, HDL cholesterol, LDL cholesterol, and triglycerides, were measured using enzymatic colorimetric methods. The analyses were performed in a certified laboratory using standardized procedures. All lipid measurements, including cholesterol and triglycerides (TG), were determined after an overnight fast.

### 2.7. Inclusion Criteria

Participants aged between 18 and 50 years old who had not undergone a cryolipolysis procedure in the past 12 months and exhibited a fat fold deemed suitable for treatment on the abdomen and flanks, as determined by the thickness of the fat layer. A minimum of 2 cm of fat in the specified regions and a BMI from 25 to 40 were required.

### 2.8. Exclusion Criteria

Pregnancy, lactation, hernia in the region, scars in the region, skin conditions, autoimmune diseases, decompensated diabetes, neoplasms, obesity above BMI > 40, paroxysmal hemoglobinuria in the cold, post-herpetic neuralgia, and cold-related diseases were grounds for exclusion, along with participants who did not meet the evaluation criteria.

### 2.9. Statistical Analysis

The participants in the study were evaluated at an initial baseline moment and then again 15 days after completing three sessions of cryolipolysis. Each participant was compared with herself before and after the treatment. For this, we used the student’s *t*-test for paired samples. The effect size through the glass delta was also calculated for the main dependent variables. Values where p was equal to or less than 0.05 were considered significant.

## 3. Results

### 3.1. Sample Characteristics

Table 1 below describes the anthropometric characteristics of the participants.

### 3.2. Body Composition Data

The body composition data obtained through the computerized tetrapolar bioimpedance assessment (BIA Tera Science) are presented in Table 2 below.

### 3.3. Absolute Fat Mass

**Figure 1:**
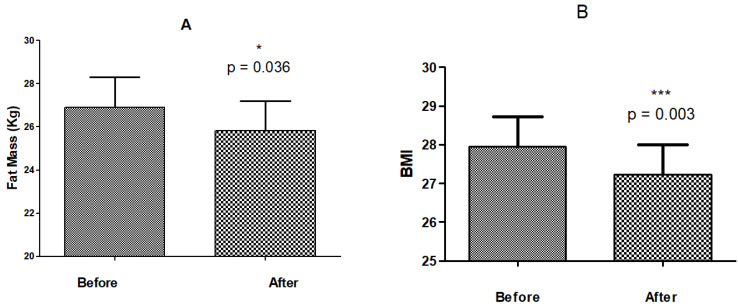
Mean and standard deviation of reduction of fat mass (kg), before and after three sessions of cryolipolysis—(**A**). Mean and standard deviation of reduction of BMI, before and after three sessions of cryolipolysis—(**B**). The *p* value was considered significant when equal to or less 0.05 N = 30 (* *p* ≤ 0.05; *** *p* ≤ 0.001).

As depicted in Figure 1A,B, the three sessions of body cold exposure treatment led to a statistically significant reduction in fat mass, from 26.9 ± 6.7 kg to 25.8 ± 6.1 kg (*p* = 0.036; paired *t*-test). Due to high intra-class variability and considering that paired analyses were performed, we also calculated the percentage change for the group. For fat mass in kilograms, an average reduction of 3.75% was observed, with a minimum of 1.4% and a maximum of 28% reduction. 

Body mass index (BMI) also presented a reduction from 27.9 ± 3.6 to 27.2 ± 3.6 (*p* = 0.0003; paired *t*-test). The reductions in fat mass and BMI represent decreases of 3.7 ± 8.7% and 2.6 ± 2.7%, respectively, both considered significant when compared before and after cryolipolysis using a paired test. We also calculated the percentage change for the group. For BMI, an average reduction of 2.61% was observed, with a minimum of 0.78% and a maximum of 10.2% of reduction.

### 3.4. Plasma Lipids

**Figure 2:**
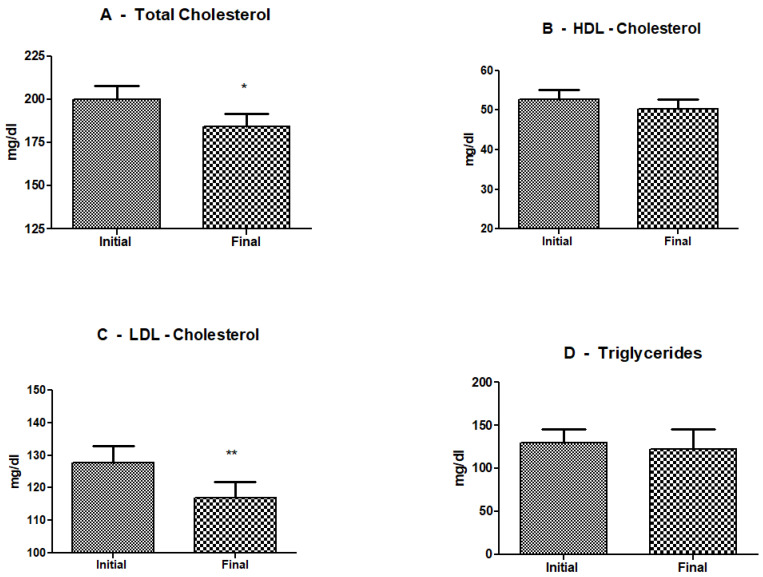
Mean and standard deviation of plasma lipids, before and after three sessions of cryolipolysis—(**A**). Mean and standard deviation of total cholesterol. (**B**) Mean and standard deviation of HDL cholesterol. (**C**) Mean and standard deviation of LDL cholesterol. (**D**) Mean and standard deviation of glycerides before and after three sessions of cryolipolysis. The *p* value was considered significant when equal to or less 0.05. N = 30, (* *p* ≤ 0.05; ** *p* ≤ 0.01).

Figure 2 illustrates the effects of three sessions of cryolipolysis on plasma lipids. In Figure 2A, it can be observed that the cold exposure of the abdominal area significantly reduced the total cholesterol levels. The average initial total cholesterol level was 199.81 mg/dL (SD = 44.34), and the average final total cholesterol level was 184.87 mg/dL (SD = 42.11). A paired t-test revealed a statistically significant reduction in total cholesterol levels post-intervention (t(29) = 3.58, *p* = 0.0012). 

Figure 2B shows that cryolipolysis did not induce any significant changes in plasma levels of HDL cholesterol. The average initial HDL cholesterol level was 52.79 mg/dL (SD = 12.19), and the average final HDL cholesterol level was 50.30 mg/dL (SD = 12.94). A paired t-test revealed that the reduction in HDL cholesterol levels post-intervention was not statistically significant (t(29) = 2.01, *p* = 0.0541). The results indicate that cryolipolysis did not significantly impact HDL cholesterol levels.

Figure 2C reveals a significant reduction in plasma levels of LDL cholesterol. The average initial LDL cholesterol level was 127.64 mg/dL (SD = 29.45), and the average final LDL cholesterol level was 116.83 mg/dL (SD = 27.06). A paired t-test revealed a statistically significant reduction in LDL cholesterol levels post-intervention (t(29) = 6.50, *p* < 0.0001). This significant decrease in LDL cholesterol levels suggests that cryolipolysis may have a beneficial impact on lipid profiles.

Figure 2D illustrates the effects of cryolipolysis on plasma triglycerides, where cold exposure did not induce any significant changes. The average initial triglyceride level was 129.30 mg/dL (SD = 60.66), and the average final triglyceride level was 123.67 mg/dL (SD = 84.69). A paired t-test revealed that the change in triglyceride levels post-intervention was not statistically significant (t(29) = 0.61, *p* = 0.5455). These results indicate that cryolipolysis did not significantly impact triglyceride levels.

**Figure 3:**
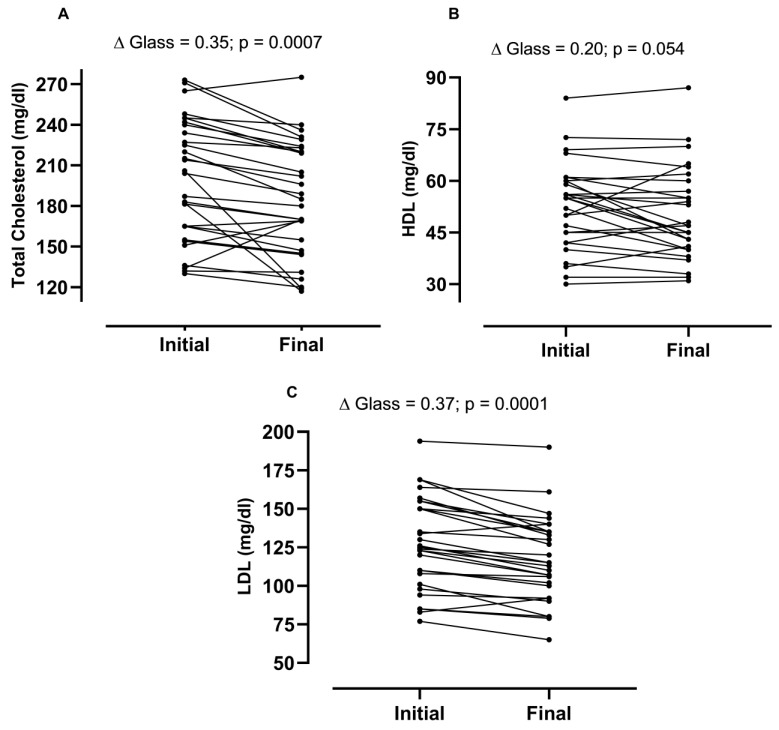
Responders and non-responders to cryolipolysis treatment. (**A**) refers to the effects of treatment on total cholesterol; (**B**) refers to the effects of treatment on HDL levels; and (**C**) refers to the effects of treatment on LDL levels, N = 30.

The results indicated significant reductions in total and LDL cholesterol levels, suggesting potential cardiovascular benefits of cryolipolysis. However, the procedure did not significantly impact HDL cholesterol or triglyceride levels. Figure 3 shows the individual data of responders and non-responders to cryolipolysis treatment.

### 3.5. Phase Angle

The results of the phase angle analysis before and after three sessions of cryolipolysis were not statistically significant. There is a tendency for an increase in phase angle values from 6.37 ± 0.57 to 6.5 ± 0.6, with a borderline *p*-value of 0.054 (paired student *t*-test).

## 4. Discussion

The results of this clinical investigation affirm the efficacy of cryolipolysis in reducing localized fat, particularly in the abdominal areas of overweight and obese women, showcasing its potential as a non-invasive alternative to surgical interventions such as liposuction. The significant decreases observed in absolute fat mass and BMI are compelling, highlighting the potential of cryolipolysis not only as a cosmetic procedure but also as a tool for improving overall health outcomes. The findings from this study illuminate the efficacy of cryolipolysis in not only reducing localized fat but also in potentially mitigating the risks associated with abdominal adiposity, particularly as they pertain to cardiovascular health and diabetes. The significant reduction in body fat and body mass index (BMI) observed post-treatment underscores cryolipolysis’s role as a compelling, non-invasive alternative to surgical fat reduction methods. More importantly, these changes in body composition were accompanied by noteworthy alterations in lipid profiles, specifically a reduction in total and LDL cholesterol, which are critical factors in the pathophysiology of metabolic and cardiovascular diseases.

The possible mechanism of fat reduction remains unclear. In addition to the classical pathway driven by sympathetic innervation and catecholamine release leading to β-receptor stimulation and lipolysis, local and direct mechanisms have been proposed. Three additional theories currently explain the damage to adipocytes (fat cells). The first, intracellular lipid crystallization, involves the formation of “lipid ice” within cells, a phenomenon initially observed in vivo on Yucatan black pigs by Manstein et al. in 2008 [22]. This process leads to direct mechanical injury to the fat cells.

The other two mechanisms relate to types of tissue injury known in organ transplantation studies: cold ischemic injury and reperfusion injury. Cold ischemic injury occurs when reduced temperature suppresses the function of the Na+-K+-ATPase, a crucial cellular osmoregulatory component, leading to changes in the electrochemical gradient and cellular edema. Additionally, the low temperatures cause significant vasoconstriction, exacerbated by the vacuum-based applicators often used in cryolipolysis treatments, which reduce blood flow and result in intracellular acidosis due to reduced metabolism and an accumulation of metabolic waste [23,24].

Reperfusion injury happens when the reintroduction of oxygen into tissues leads to the formation of reactive oxygen species from ATP degradation products, further damaging cells. Together, these mechanisms are thought to lead to the activation of apoptotic pathways, resulting in the death of adipocytes and a localized inflammatory response. Histopathological evaluations post-treatment have shown increased macrophage activity in the inflamed areas, suggesting their role in recycling dead adipocytes and supporting the mechanism by which cryolipolysis does not significantly raise serum lipid levels or the levels of lipid-associated proteins and enzymes [25].

While the current theoretical framework is physiologically plausible and supported by preliminary studies, it largely relies on early animal research and ex vivo experiments. There is a recognized need for more extensive and rigorous examination of these mechanisms in clinical settings to fully understand the efficacy and implications of cryolipolysis technology.

The reduction in total cholesterol observed in this study is particularly significant given the strong link between excessive abdominal fat and increased cardiovascular risk. Abdel-Aa in 2020 [26] first demonstrated that women who participated in a combination of cryolipolysis and a diet program experienced more significant improvements in lipid profiles and liver enzyme levels compared to those who followed only a diet program. The study found a significant decrease in total and LDL cholesterol. Interventions aimed at reducing waist circumference, coupled with the decrease in abdominal subcutaneous fat, amplified the overall systemic benefits of cryolipolysis. Previously, Zelickson et al. [27]. observed that treating pigs (N = 3) led to a notable decrease in the superficial fat layer without harming the overlying skin. This reduction was preceded by an inflammatory response, initiated by the cold-induced apoptosis of adipocytes. Furthermore, an evaluation of lipid levels over a three-month period post-treatment revealed that cholesterol and triglyceride values remained within the normal range. In this case, a significant limitation of the study was that it only involved three pigs. In contrast, our study included 30 women who underwent three sessions of cryolipolysis, with all parameters evaluated before and after the treatments for each participant, using a paired comparison approach. 

Abdominal fat is metabolically active and contributes to elevated levels of circulating lipids, which are pivotal in the development of atherosclerosis. By reducing total cholesterol, cryolipolysis may reduce the burden of cholesterol plaques within the arteries, thereby potentially lowering the risk of heart attacks and strokes. This is of paramount importance in a clinical setting, as cardiovascular diseases remain the leading cause of mortality globally.

The pronounced reduction in LDL cholesterol further highlights the potential of cryolipolysis to improve arterial health. LDL particles are known to infiltrate the endothelial layer of arteries, instigating an inflammatory response that leads to the development of atherosclerotic plaques. The ability of cryolipolysis to significantly decrease LDL cholesterol suggests that it may help in staving off the cascade of events leading to atherosclerosis and subsequent cardiovascular events. This result was previously observed [26] when cold exposure was able to reduce plasma lipids better than diet. However, the observed decrease in cholesterol could also be influenced by dietary factors or the use of cholesterol for the construction of cell membranes during tissue recovery post-cryoipolysis. Further studies are needed to isolate the effects of cryolipolysis from dietary influences.

Despite these promising results, it is important to note that cryolipolysis did not significantly alter the levels of HDL cholesterol or plasma triglycerides. This indicates that while the procedure can influence certain lipid fractions, it does not uniformly affect all aspects of the lipid profile. These findings highlight the complexity of lipid metabolism and suggest that individual responses to cryolipolysis may vary, necessitating personalized treatment approaches.

Concerning another important problem related to body fat, the epidemic proportions of type 2 diabetes worldwide and implications of reducing central adiposity extend beyond cardiovascular diseases. Abdominal fat is a known risk factor for insulin resistance, the hallmark of type 2 diabetes. By diminishing abdominal fat, cryolipolysis could play a role in improving insulin sensitivity, thereby contributing to diabetes management and prevention. Although this study did not measure insulin sensitivity directly, the reductions in fat mass and improvements in lipid profiles may indirectly suggest improvements in metabolic health. Recently, Mazor et al. [28], also working with pigs (N = 8), demonstrated that mesenteric fat cryolipolysis (MFC) safely reduced mesenteric fat volume by 30% at three months, a reduction that was sustained at six months. There were no changes in body weight in either the MFC or the sham surgery control groups. Additionally, measures of glycemic control, insulin sensitivity, and blood pressure showed significant improvements in the MFC group compared to the sham controls.

Visceral fat, located deep within the abdominal cavity and surrounding vital organs, is particularly implicated in the pathogenesis of metabolic syndrome, a cluster of conditions that significantly increase the risk of cardiovascular disease, diabetes, and stroke. Unlike subcutaneous fat, which is distributed just beneath the skin, visceral fat is metabolically active and secretes a variety of adipokines and inflammatory markers that contribute to insulin resistance, a core component of metabolic syndrome [29]. The presence of excess visceral fat disrupts normal lipid metabolism and hormone functions, leading to elevated glucose and insulin levels, increased blood pressure, and abnormal cholesterol levels, notably raised triglycerides and reduced high-density lipoprotein (HDL) cholesterol [29]. The relationship between visceral fat and these metabolic disturbances underscores its role as a critical mediator of metabolic risk factors. Moreover, the distribution of body fat is a significant determinant of metabolic health. Central obesity, characterized by increased abdominal fat, is a well-established risk factor for metabolic syndrome and its associated conditions [30]. This form of obesity is closely associated with greater visceral fat accumulation, which is more detrimental than peripheral or subcutaneous fat deposition. Studies have shown that individuals with central obesity are at a higher risk of developing insulin resistance, hypertension, and dyslipidemia, which are precursors to more severe metabolic and cardiovascular diseases. Effective management of body fat, particularly through interventions that target visceral fat reduction, such as cryolipolysis, may offer therapeutic benefits in mitigating the onset and progression of metabolic syndrome by improving body fat distribution and reducing the pro-inflammatory state associated with excessive visceral fat. Here we used the waist perimeter as an indicator of abdominal fat. Waist perimetry was chosen as a simple and reliable measure of abdominal obesity. However, we should say that including other anthropometric measures such as Waist-to-Hip Ratio (WHR), conicity index, and skinfold thickness would provide a more comprehensive assessment of body composition. Future studies should incorporate these additional measures to strengthen the scientific rigor of the findings.

The regulation of triglyceride (TAG) stores in adipocytes is tightly controlled by hormonal factors, primarily influenced by catecholamines such as norepinephrine. Released in response to cold, norepinephrine activates β-adrenergic receptors (β-ARs), which stimulate adenylyl cyclase to increase cyclic AMP (cAMP) levels. This elevation in cAMP leads to the activation of protein kinase A (PKA), which plays a crucial role in lipolysis by phosphorylating proteins such as perilipin 1 (PLIN1) and hormone-sensitive lipase (HSL). PKA phosphorylation facilitates the mobilization of co-activators and enzymes essential for breaking down TAGs into diacylglycerols (DAGs) and further into free fatty acids [31]. Notably, recent research suggests that direct activation of adipose triglyceride lipase (PNPLA2) may be sufficient for lipolysis without PKA stimulation, indicating a potential redundancy in the pathways that mediate fat cell lipolysis. Additionally, PKA is known to phosphorylate other proteins such as PLIN5, enhancing lipolysis further. This complex regulation underscores the intricate hormonal control of fat metabolism, particularly in response to cold exposure, which activates non-shivering thermogenesis. Physiological stimulation of brown fat β3-adrenergic receptors (β3-AR) through cold stress, or through direct pharmacological activation swiftly triggers non-shivering thermogenesis. This process is facilitated by the action of mobilized fatty acids, which serve as allosteric activators of uncoupling protein 1 (UCP1), the key molecular mechanism responsible for heat production in brown fat [32,33].

### Phase Angle and Metabolic Health

The increase in phase angle values reported in this study suggests improvements in cell membrane integrity and function, which could be indicative of enhanced cellular health and metabolic efficiency. A higher phase therapeutic implications of cryolipolysis in broader metabolic contexts necessitate further investigations into how changes in body composition affect cellular and systemic metabolic functions [34].

Future studies should focus on the long-term metabolic outcomes of cryolipolysis, particularly exploring its effects on insulin resistance, glucose homeostasis, and inflammatory markers, which are crucial in the pathogenesis of both cardiovascular diseases and diabetes. Additionally, investigating the differential impact of cryolipolysis on various demographic groups could elucidate factors that influence treatment efficacy, paving the way for personalized therapeutic strategies.

## 5. Conclusions

In conclusion, this study positions cryolipolysis as a promising non-invasive treatment modality not only for cosmetic fat reduction but also as a potential therapeutic intervention in the management and prevention of metabolic diseases. Integrating cryolipolysis with broader lifestyle and dietary interventions could potentially enhance its efficacy and contribute to comprehensive metabolic health management. However, it is imperative to approach these findings with caution until further robust clinical trials are conducted to substantiate these potential health benefits.

## Figures and Tables

**Table 1:** Anthropometric characteristics of the participants.

Anthropometric Characteristics			
	Average	STDV	SE
Body Weight (kg)	73.14	10.8	2.01
Height (cm)	163	4.37	0.81
Age (Years)	40.8	11.4	2.11
Body Mass Index (kg/m^2^)	27.5	3.87	0.72

**Table 2:** Body values of the participants before and after cryolipolysis treatments.

Cryolipolysis Treatment				
	Before	After	*p* Value	∆ Glass
Lean Mass (kg)	48.0 ± 4.8	47.0 ± 4.1		
Absolute Fat Mass (kg)	26.9 ± 6.7	25.8 ± 6.0	0.036	0.16
Total Water (L)	34.2 ± 3.9	33.6 ± 3.4		
Intracellular Water (L)	17.8 ± 1.6	17.5 ± 3.8		
Extracellular Water (L)	16.4 ± 2.2	16.1 ± 1.8		
Body Mass Index	27.9 ± 3.7	27.2 ± 3.5	0.003	0.18
Phase Angle	6.4 ± 0.5	6.5 ± 0.6	0.054	0.20

## Data Availability

The original contributions presented in the study are included in the article, further inquiries can be directed to the corresponding author.
